# Michaelis-like complex of SARS-CoV-2 main protease visualized by room-temperature X-ray crystallography

**DOI:** 10.1107/S2052252521010113

**Published:** 2021-10-05

**Authors:** Daniel W. Kneller, Qiu Zhang, Leighton Coates, John M. Louis, Andrey Kovalevsky

**Affiliations:** a Neutron Scattering Division, Oak Ridge National Laboratory, 1 Bethel Valley Road, Oak Ridge, TN 37831, USA; b National Virtual Biotechnology Laboratory, US Department of Energy, Washington, DC 20585, USA; c Second Target Station, Oak Ridge National Laboratory, 1 Bethel Valley Road, Oak Ridge, TN 37831, USA; d Laboratory of Chemical Physics, National Institute of Diabetes and Digestive and Kidney Diseases, National Institutes of Health, DHHS, Bethesda, MD 20892-0520, USA

**Keywords:** SARS-CoV-2, 3CL protease, main protease, catalytic mechanism, C145A mutant, enzyme–substrate complex, room-temperature X-ray crystallography

## Abstract

SARS-CoV-2 emerged at the end of 2019 to cause an unprecedented pandemic of the deadly respiratory disease COVID-19 that continues to date. The viral main protease (M^pro^) is essential for SARS-CoV-2 replication and is therefore an important drug target. Understanding the catalytic mechanism of M^pro^, a cysteine protease with a catalytic site comprising the noncanonical Cys145–His41 dyad, can help in guiding drug design. Here, a 2.0 Å resolution room-temperature X-ray crystal structure is reported of a Michaelis-like complex of M^pro^ harboring a single inactivating mutation C145A bound to the octapeptide Ac-SAVLQSGF-CONH_2_ corresponding to the nsp4/nsp5 autocleavage site. The peptide substrate is unambiguously defined in subsites S5 to S3′ by strong electron density. Superposition of the Michaelis-like complex with the neutron structure of substrate-free M^pro^ demonstrates that the catalytic site is inherently pre-organized for catalysis prior to substrate binding. Induced fit to the substrate is driven by P1 Gln binding in the predetermined subsite S1 and rearrangement of subsite S2 to accommodate P2 Leu. The Michaelis-like complex structure is ideal for *in silico* modeling of the SARS-CoV-2 M^pro^ catalytic mechanism.

## Abstract 2

Understanding the catalytic mechanism of SARS-CoV-2 main protease (M^pro^) can help in guiding drug design of specific small-molecule antivirals. A 2.0 Å resolution room-temperature X-ray crystal structure of inactive C145A mutant M^pro^ in complex with the octapeptide Ac-SAVLQSGF-CONH_2_ that resembles a Michaelis complex is reported.

## 1. Introduction   

Severe acute respiratory syndrome coronavirus 2 (SARS-CoV-2) causes the deadly coronavirus disease 2019 (COVID-19) that engulfed the globe in a pandemic in early 2020 and remains a significant health threat despite the availability of several vaccines and antiviral therapies (Gavor *et al.*, 2020 ▸; Meo *et al.*, 2021 ▸; Doroftei *et al.*, 2021 ▸; Bidram *et al.*, 2021 ▸). The main protease of SARS-CoV-2 (3CL^pro^ or M^pro^) is virally encoded and its precise function is essential for virus replication. M^pro^ cleaves two polyproteins, pp1a and pp1ab (∼450 and ∼790 kDa, respectively), into individual functional proteins that build the virus genome replication/transcription machinery (Wu *et al.*, 2020 ▸; Xu *et al.*, 2020 ▸). M^pro^ is a cysteine protease (Tong, 2002 ▸) that hydrolyses scissile peptide bonds at 11 specific locations, including its own release as the non­structural protein nsp5 (termed autoprocessing), in the polyprotein precursors. The substrate specificity of M^pro^ and its active-site architecture are dissimilar relative to human proteases. Thus, M^pro^ is considered an important drug target for the design and development of specific antivirals with potentially minimal nonspecific binding to human proteins (Liu *et al.*, 2020 ▸; Jin *et al.*, 2020 ▸; Ton *et al.*, 2020 ▸; Suárez & Díaz, 2020 ▸; Acharya *et al.*, 2020 ▸; Pathak *et al.*, 2021 ▸), including the possibility of designing pan-coronavirus antivirals (Ullrich & Nitsche, 2020 ▸).

M^pro^ is a homodimer, with each protomer composed of three distinct domains (Zhang *et al.*, 2020 ▸; Jin *et al.*, 2020 ▸; Anand *et al.*, 2002 ▸). The catalytic domains I (residues 1–101) and II (residues 102–200) have antiparallel β-barrel structures that resemble the chymotrypsin fold. The helical domain III (residues 201–306) comprises five α-helices and promotes dimerization through interactions with specific residues of domains I and II (Chou *et al.*, 2004 ▸; Barrila *et al.*, 2006 ▸; Tsai *et al.*, 2010 ▸). The catalytic site of M^pro^ is non­canonical; it has a catalytic Cys145–His41 dyad believed to be assisted by a conserved (catalytic) water molecule (Wang *et al.*, 2020 ▸), instead of the more common (Ser)Cys–His–Asp(Glu) triads found in other cysteine (and serine) proteases (Gorbalenya & Snijder, 1996 ▸). The M^pro^ cleavage sites are characterized by a preference for Gln and Leu/Phe at substrate positions P1 and P2, respectively (Fan *et al.*, 2004 ▸; Goetz *et al.*, 2007 ▸). Substrate specificity is conserved in most coronavirus M^pro^ cleavage sites (Lai *et al.*, 2006 ▸). Based on the crystal structures of the closely related SARS-CoV M^pro^ inactive mutant H41A in complex with an 11-amino-acid peptide substrate (Xue *et al.*, 2008 ▸) and SARS-CoV M^pro^ inactive mutant C145A containing a ten-residue C-terminal prosequence (Muramatsu *et al.*, 2016 ▸), the M^pro^ active-site cavity located on the protein surface can accommodate up to nine substrate residues in positions P5 through P4′ in the corresponding substrate-binding subsites S5 through S4′.

To date, most of the SARS-CoV-2 M^pro^ X-ray crystal structures deposited in the Protein Data Bank (PDB) and associated studies have focused on the design of protease inhibitors. Conversely, there is an apparent lack of structural studies into the catalytic mechanism of SARS-CoV-2 M^pro^. Lee *et al.* (2020 ▸) have recently reported capturing a covalent acyl-enzyme intermediate using native enzyme and a product complex using the inactive C145A mutant. In both structures, the C-terminal residues of one protomer extend into the active site of the other protomer, thus mimicking autocleavage of its C-terminus. Consequently, a Michaelis-like complex structure of SARS-CoV-2 M^pro^ bound to a discrete peptide substrate remains unidentified. This paper reports a room-temperature X-ray crystal structure of SARS-CoV-2 M^pro^ C145A mutant (M^pro/C145A^) in complex with the octapeptide Ac-SAVLQSGF-CONH_2_ corresponding to the nsp4/nsp5 autocleavage site (henceforth referred to as M^pro/C145A^–substrate), at 2.0 Å resolution and pH 6.5. The peptide substrate spans residues from P5 through P3′ and is chemically protected by acetylation at the N-terminus and amidation at the C-terminus. The octapeptide binds asymmetrically in the M^pro^ active-site cavity such that the five groups P5–P1 bind to the nonprime substrate-binding subsites S5 through S1 and the three groups P1′–P3′ bind to the prime substrate-binding subsites S1′ through S3′. We hypothesize that this substrate mode of binding is the reason behind our success in producing crystals of such a complex. We previously reported enzyme kinetics measurements for a FRET substrate based on the same nsp4/nsp5 autocleavage site (*k*_cat_ = 0.28 s^−1^ and *K*_m_ = 170 µ*M*; Kneller, Galanie *et al.*, 2020 ▸).

## 2. Materials and methods   

### 2.1. General information   

Crystallization reagents and supplies were purchased from Hampton Research, Aliso Viejo, California, USA. Crystallographic supplies for crystal mounting and X-ray diffraction data collection at room temperature were purchased from MiTeGen, Ithaca, New York, USA. The Ac-SAVLQSGF-CONH_2_ peptide used in this study was custom-synthesized by Biomatik Corp., Kitchener, Ontario, Canada.

### 2.2. Expression and purification of M^pro/C145A^   

A DNA insert encoding a 6×His tag followed by a TEV protease cleavage site and SARS CoV-2 M^pro^ (306 amino acids) bearing an active-site C145A mutation was cloned into the pJ414 vector (ATUM) and transformed into *Escherichia coli* BL21(DE3) cells (Agilent). The last residue in the TEV protease recognition sequence (ENLYFQG/S) was chosen to be a serine, such that upon cleavage the M^pro/C145A^ construct starts with the native N-terminal Ser1 residue. Cells were grown in Luria–Bertani medium, and the M^pro/C145A^ fusion protein was expressed using established protocols. The fusion protein was purified under native conditions by lysis and Ni–NTA affinity chromatography in 20 m*M* Tris–HCl pH 8, 150 m*M* NaCl, 1 m*M* TCEP, 20 m*M* imidazole. This step was followed by size-exclusion chromatography on a Superose 12 column (16 × 60 cm, Cytiva) to remove minor high-molecular-weight contaminants and to exchange the buffer to 25 m*M* Tris–HCl pH 8, 100 m*M* NaCl, 0.5 m*M* EDTA, 1 m*M* DTT, 20 m*M* imidazole, which is suitable for cleavage by TEV protease at a molar ratio of 75:1 fusion protein:TEV protease. TEV protease was prepared as described by Lucast *et al.* (2001 ▸). Upon completion of the cleavage and adjustment of the EDTA concentration to 0.25 m*M*, M^pro/C145A^ was collected in the flowthrough from the Ni–NTA column, concentrated and then subjected again to isocratic fractionation on Superose 12 in 25 m*M* Tris pH 7.6, 150 m*M* NaCl, 1 m*M* TCEP. Peak fractions were combined, concentrated and aliquots of the protein were stored at − 20 C. Purity was verified both by SDS–PAGE and electrospray ionization mass spectrometry.

### 2.3. Crystallography of the M^pro/C145A^–substrate complex   

We have previously published detailed methods for crystallizing high-quality M^pro^ crystals using the sitting-drop vapor-diffusion methodology (Kneller, Galanie *et al.*, 2020 ▸; Kneller, Phillips, Kovalevsky *et al.*, 2020 ▸). Crystallization conditions for the M^pro/C145A^ mutant were discovered by automated high-throughput screening at the Hauptman–Woodward Medical Research Institute (Luft *et al.*, 2003 ▸). The protein was concentrated to 6 mg ml^−1^. Initially, flower-like crystal aggregates unsuitable for room-temperature X-ray diffraction grew using a sample of 270 µl protein solution mixed with 1.6 mg lyophilized peptide in 20% PEG 3350, 0.1 *M* bis-Tris pH 7.0. Subsequently, the protein was mixed in a 1:5 molar ratio with peptide from a 50 m*M* stock in 100% DMSO and used for crystallization with 20% PEG 3350, 0.1 *M* bis-Tris pH 6.5. This condition produced a final pH in the crystallization drop of 6.5 as measured by a microelectrode. Drops were seeded using a cat whisker and flower-shaped aggregates grew with thicker ‘petal’ substituents after one week of incubation at 14°C. A single crystal from the aggregate was harvested and mounted on loops and capillary setups for room-temperature diffraction.

### 2.4. Room-temperature X-ray diffraction data collection and structure refinement   

Room-temperature X-ray diffraction data were collected on a Rigaku HighFlux HomeLab instrument equipped with a MicroMax-007 HF X-ray generator, Osmic VariMax optics and a Dectris EIGER R 4M hybrid photon-counting detector. Data were integrated using the *CrysAlis^Pro^* software suite (Rigaku, The Woodlands, Texas, USA) and were reduced and scaled using *AIMLESS* (Evans & Murshudov, 2013 ▸) from the *CCP*4 suite (Winn *et al.*, 2011 ▸). The M^pro/C145A^–substrate structure was solved by molecular replacement using PDB entry 6wqf (Kneller, Phillips, O’Neill *et al.*, 2020 ▸) with *Phaser* (McCoy *et al.*, 2007 ▸) from *CCP*4. The model was iteratively refined using *phenix.refine* from the *Phenix* suite (Liebschner *et al.*, 2019 ▸) and *Coot* (Emsley *et al.*, 2010 ▸; Casañal *et al.*, 2020 ▸) aided by *MolProbity* (Chen, Arendall *et al.*, 2010 ▸) for geometry validation. Final data-collection and refinement statistics are given in Supplementary Table S1.

## 3. Results and discussion   

### 3.1. Binding of peptide substrate   

**Figure 1:**
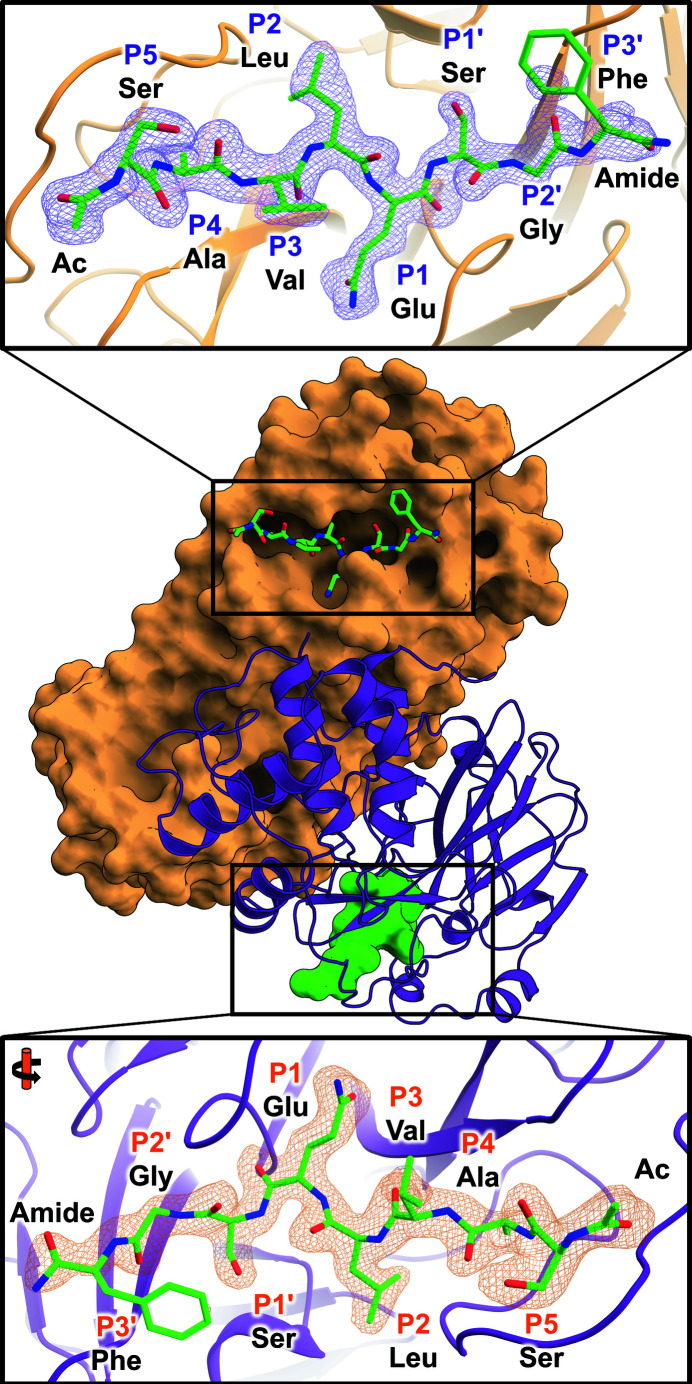
M^pro/C145A^–substrate complex. M^pro/C145A^ (protomer *A* is shown as a purple cartoon and protomer *B* as an orange surface) was co-crystallized with an octapeptide (green sticks and surface) corresponding to the nsp4/nsp5 autocleavage site. The top inset shows the peptide in protomer *B* in conventional orientation, while the bottom inset shows protomer *A* rotated by 180°. *F*_o_ − *F*_c_ omit difference electron-density maps for each peptide are contoured at 3σ.

**Figure 2:**
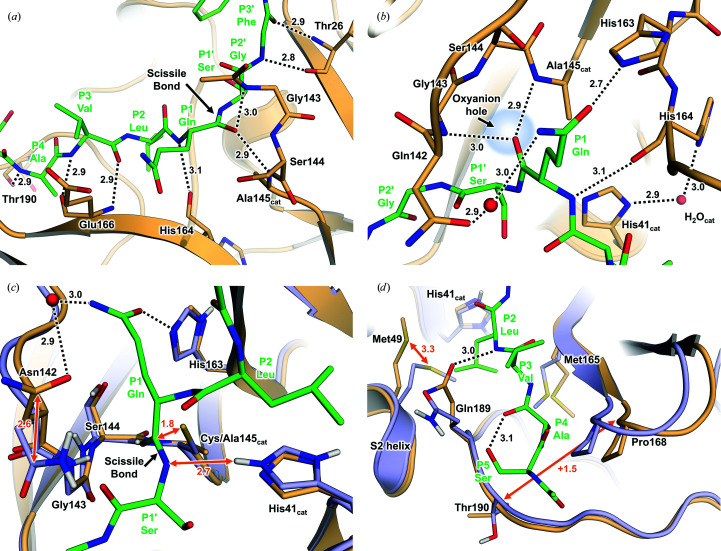
M^pro/C145A^–substrate interactions for induced fit of substrate. (*a*) Main-chain hydrogen-bond interactions between M^pro^ and the peptide substrate (shown as orange and green C atoms, respectively). Hydrogen bonds are shown as dotted lines. Distances are in Å. (*b*) Hydrogen-bond interactions between peptide P1 Gln with the S1 subsite and the catalytic site. Water molecules are shown as red spheres. (*c*) Superposition of M^pro/C145A^–substrate with the substrate-free M^pro^ neutron crystal structure (light blue; PDB entry 7jun) showing the scissile bond in relation to the catalytic Cys145–His41 zwitterion. Superposition was by a least-squares fit to C^α^ atoms of protomer *A*. (*d*) Induced fit of M^pro/C145A^ to the peptide substrate by the rearrangements of side chains in the S2 and S4 subsite.

In M^pro/C145A^–substrate, a substrate occupies each of the two active-site grooves in the M^pro^ homodimer structure (Fig. 1 ▸). There is unambiguous electron density for all residues except for the P3′ Phe side chain. Interactions between M^pro^ and substrate are nearly identical in both protomers; hence, subsequent details of our analysis will focus on protomer *A*. Main-chain interactions between the peptide and M^pro^ are consistent with adding a β-strand to the antiparallel β-sheets flanking the scissile bond [Fig. 2 ▸(*a*)]. There are eight hydrogen bonds between the main chains of the peptide substrate and M^pro^, which involve residues Thr190, Glu166, His164, Ser144, Gly143 and Thr26. In the catalytic site, P1 Gln is driven into the S1 subsite via a 2.7 Å hydrogen bond to the His163 imidazole and a water-mediated interaction with Asn142, whereas its main-chain carbonyl O atom is centered into the characteristic oxyanion hole [Fig. 2 ▸(*b*)]. The bulky hydrophobic P2 Leu side chain fills its respective hydrophobic S2 subsite, whereas the smaller P4 Ala juts into the shallow S4 pocket capped by the flexible S5 loop. P2′ Gly contributes to substrate binding via backbone hydrogen-bond interactions with Thr26, whereas P1′ Ser makes no hydrogen bonds to M^pro^, as is the case for P5 Ser. The P5 Ser, P3 Val, P2′ Ser and P3′ Phe side chains face the solvent space between crystallo­graphic symmetry-related molecules, with the P3′ Phe phenyl ring being dynamic and poorly defined in the electron-density maps.

The C-terminal residues of M^pro^ flank the last helix in domain III. Here, residues 302–306 constitute a flexible loop (Kneller, Phillips, O’Neill *et al.*, 2020 ▸) and are typically disordered in crystal structures of SARS-CoV-2 M^pro^ to the extent that some crystallographers choose not to model the C-terminus (Dai *et al.*, 2020 ▸; Zhang *et al.*, 2020 ▸). In this context, it is noteworthy that electron density for the C-terminal residues up to the very last Gln306 amine is clearly observed in M^pro/C145A^–substrate (Supplementary Fig. S1). When the C-terminal residues of M^pro/C145A^–substrate and the product complex reported by Lee *et al.* (2020 ▸) are superimposed, the main-chain atoms align with an r.m.s.d. of 0.7 Å for residues 303–306 (Supplementary Fig. S2), demonstrating that the C-terminus of M^pro^ is pre-organized to bind into the active site during M^pro^ autoprocessing.

### 3.2. Comparison with substrate-free M^pro^   

Superposition of M^pro/C145A^–substrate and our recently determined substrate-free neutron crystallographic structure of wild-type SARS-CoV-2 M^pro^ (PDB entry 7jun; Kneller, Phillips, Weiss *et al.*, 2020 ▸) offers unique insights into catalysis because the Cys145–His41 dyad was observed as a zwitterionic species with the Cys145 thiolate S^γ^ atom 3.8 Å away from His41 N^ɛ2^, already primed for proteolysis [Fig. 2 ▸(*c*)]. When the two structures are superimposed, the Cys145 thiolate is positioned 1.8 Å away from the scissile-bond carbonyl C atom with an ideal distance and orientation for a nucleophilic attack. The scissile-bond amide N atom is positioned 2.7 Å away from the N^ɛ2^ hydrogen on the catalytic His41, which does not significantly change its position in the complex, poised for proton transfer to the amino-terminus that is created in the cleavage reaction. The M^pro/C145A^–substrate structure therefore underscores the suggestion that the zwitterionic catalytic dyad is present prior to substrate binding (Kneller, Phillips, Weiss *et al.*, 2020 ▸) instead of requiring reorganization of the catalytic site to facilitate proton transfer from Cys145 to His41 in the catalytically resting (nonreactive) state (*i.e.* a protonated Cys145 thiol and singly protonated His41 imidazole) to create the reactive zwitterionic species (Paasche *et al.*, 2014 ▸; Ramos-Guzmán *et al.*, 2020 ▸; Díaz & Suárez, 2021 ▸).

In subsite S1, Glu166 accommodates P1 Gln by moving ∼0.9 Å away from the substrate and simultaneously rotating to form a 2.8 Å hydrogen bond to His172 that is absent in substrate-free M^pro^. This conformational change of the Glu166 side chain is due to the binding of a ligand to the active site and is consistent with the changes observed in M^pro^–inhibitor complexes (Kneller, Galanie *et al.*, 2020 ▸; Dai *et al.*, 2020 ▸). Meanwhile, the Asn142 side chain flips ∼180° and shifts 2.6 Å from its position in the substrate-free structure to produce water-mediated contacts with P1 Gln. This comparison indicates that the catalytic dyad is already pre-organized and primed for catalysis in the substrate-free structure, requiring minimal rearrangements to initiate the peptide-hydrolysis reaction. Induced fit of the peptide substrate to M^pro^ is achieved primarily through the continuation of β-sheet structures and key rearrangements of the large side chains Met165, Met49 and Gln189 from their positions in substrate-free M^pro^ to create subsites S2 and S4 [Fig. 2 ▸(*d*)]. The substrate P2 Leu forms the S2 subsite by a >3 Å steric swing of the Met49 side chain located on the short S2 helix (residues 46–50). P2 Leu also acts in consortium with the methyl C atom of P4 Ala to produce the shallow S4 subsite by initiating a new rotational isomer for Met165 and shifting the position of the S4 β-hairpin loop by as much as 1.5 Å. The peptide residue arrangement is stabilized by Gln189 rotating to hydrogen-bond to the main-chain NH of P3 Val. P5 Ser does not interact directly with M^pro^. However, the hydroxymethyl side chain might act to nullify repulsion of the Gln189 backbone by intramolecular hydrogen bonding to the carbonyl of P3 Val. The active site around P4 and P5 expands by >1.5 Å from the substrate-free structure to accommodate the substrate, as measured by the C^α^ distances between Pro168/168′ and Thr190/190′. Interestingly, the S2 subsite helix of the complex shows only minor distal expansion, unlike in M^pro^–inhibitor complexes where the S2 subsite helix can shift by >1.5 Å (Jin *et al.*, 2020 ▸; Kneller, Galanie *et al.*, 2020 ▸). Similarly, the S5 loop (residues 189–194) is only minimally disturbed by the substrate, shifting by <1.0 Å.

### 3.3. Comparison with SARS-CoV M^pro/H41A^   

**Figure 3:**
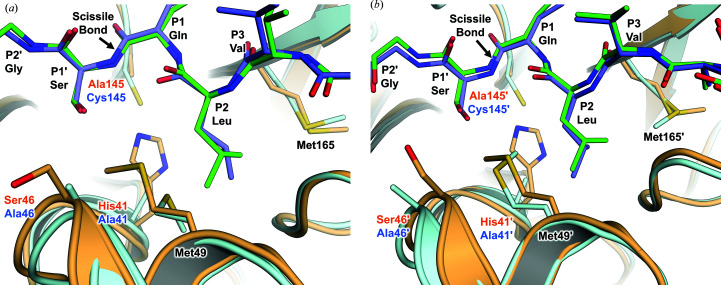
Comparison of substrate-bound M^pro/C145A^ from SARS-CoV-2 (PDB entry 7n89) with M^pro/H41A^ from SARS-CoV (PDB entry 2q6g). (*a*) Protomer *A* in M^pro/C145A^–substrate from SARS-CoV-2 (orange protein, green substrate) superimposed on the SARS-CoV M^pro/H41A^–substrate complex (cyan protein, slate substrate). Superposition was by a least-squares fit of C^α^ atoms of protomer *B* in PDB entry 2q6g to protomer *A* in PDB entry 7n89. (*b*) Protomer *B* in M^pro/C145A^–substrate from SARS-CoV-2 (orange protein, green substrate) superimposed on the SARS-CoV M^pro/H41A^–substrate complex. Superposition was by a least-squares fit of C^α^ atoms of protomer *A* in PDB entry 2q6g to protomer *B* in PDB entry 7n89.

It is instructive to compare our room-temperature M^pro/C145A^–substrate structure from SARS-CoV-2 with a low-temperature structure of the SARS-CoV main protease H41A mutant, M^pro/H41A^, soaked with an 11-amino-acid peptide substrate spanning the same nsp4/nsp5 autocleavage site (Xue *et al.*, 2008 ▸; Fig. 3 ▸; PDB entry 2q6g). The peptide substrate in both structures bows into the active site by conforming to the antiparallel β-sheets flanking the catalytic site. Overall, the orientations of the substrate residues in both protomers are in agreement or can be explained by a lack of electron density, with one exception. The C^γ^ atoms of P2 Leu deviate by ∼1 Å, which is coordinated with the rotational isomer differences observed for the Met49 and Met165 side chains. In the SARS-CoV M^pro/H41A^–substrate complex, the substrate pushes the catalytic Cys145 side chain away from the scissile bond towards the vacated space left by the H41A mutation to a position where the S atom is 4.5 Å from the scissile carbonyl C atom. M^pro^ possesses 96% sequence identity between SARS-CoV and SARS-CoV-2. The nearest mutation to the active site in SARS-CoV-2 M^pro^, A46S, does not introduce appreciable differences despite minor differences in the S2 subsite helix.

## 4. Conclusion   

We describe details of the co-crystal structure of SARS-CoV-2 M^pro^ bearing an active-site C145A mutation in complex with an octapeptide substrate corresponding to the natural nsp4/nsp5 junction sequence which M^pro^ autoprocesses prior to its C-terminal junction (nsp5/nsp6 site) during its maturation from the polyprotein precursor (Hsu *et al.*, 2005 ▸; Chen, Jonas *et al.*, 2010 ▸; Muramatsu *et al.*, 2013 ▸). Substrate affinity is driven primarily by P1 Gln hydrogen-bonding interactions and P2 Leu induced-fit rearrangement, with assistance from secondary-structure main-chain interactions. Superposition of the substrate complex with the substrate-free M^pro^ neutron structure reinforces the suggestion that the substrate-free M^pro^ structure is innately pre-organized to attack the scissile bond upon a substrate-binding event. We also suggest that our structure is an ideal starting point for *in silico* modeling and simulations looking into understanding M^pro^–substrate relationships due to specificity and the absence of cryo-artifacts. These results bring timely insights into the nature of catalysis to expedite structure/mechanism-based drug design.

## Supplementary Material

PDB reference: room-temperature X-ray crystal structure of SARS-CoV-2 main protease C145A mutant in complex with the substrate Ac-SAVLQSGF-CONH_2_, 7n89

Supplementary Table and Figures. DOI: 10.1107/S2052252521010113/mf5056sup1.pdf
