# Supplementary material for: Michaelis-like complex of SARS-CoV-2 main protease visualized by room-temperature X-ray crystallography
Source: IUCrJ. 2021 Oct 5;8(Pt 6):973–9. doi: 10.1107/S2052252521010113 (PMC8562657; doi:10.1107/S2052252521010113)
Supplement: Supplementary file 1 [file m-08-00973-sup1.pdf]

IUCrJ

Volume 8 (2021)

Supporting information for article:

Michaelis-like complex of SARS-CoV-2 main protease visualized by room-temperature X-ray crystallography

Daniel W. Kneller, Qiu Zhang, Leighton Coates, John M. Louis and Andrey Kovalevsky

Table S1. Crystallographic data collection and refinement statistics for the room-temperature X-ray structure of SARS-CoV-2 $M^{pro/C145A}$ in complex with a peptide substrate corresponding to the nsp4/nsp5 autocleavage site. Values in parentheses are for the highest-resolution shell.

	**$M^{pro/C145A}$-Substrate** (293K) PDB ID 7N89
**Data collection:**	**X-ray (in-house)**
Diffractometer	Rigaku HighFlux Eiger 4M
Space group	P1
Wavelength (Å)	1.5406
Cell dimensions:	
a, *b*, *c* (Å)	46.65, 54.10, 60.50
a, b, g (°)	66.88, 79.02, 88.52
Resolution (Å)	Inf-2.00 (2.08-2.00)
No. reflections unique	34481 (3449)
R_{merge}	0.103 (0.361)
R_{pim}	0.062 (0.332)
$CC_{1/2}$	0.988 (0.715)
$I / \sigma I$	8.20 (2.19)
Completeness (%)	93.6 (87.9)
Redundancy	2.3 (2.3)
**Refinement:**	
R_{work} / R_{free}	0.1706 / 0.2180
B-factors	
Protein	27.85
Substrate	28.62
Water	33.41
Ramachandran statistics	
Favored (%)	97.75
Allowed (%)	2.25
Outliers (%)	0
R.M.S. deviations	
Bond lengths (Å)	0.003
Bond angles (°)	0.583
All atom clashscore	2.19

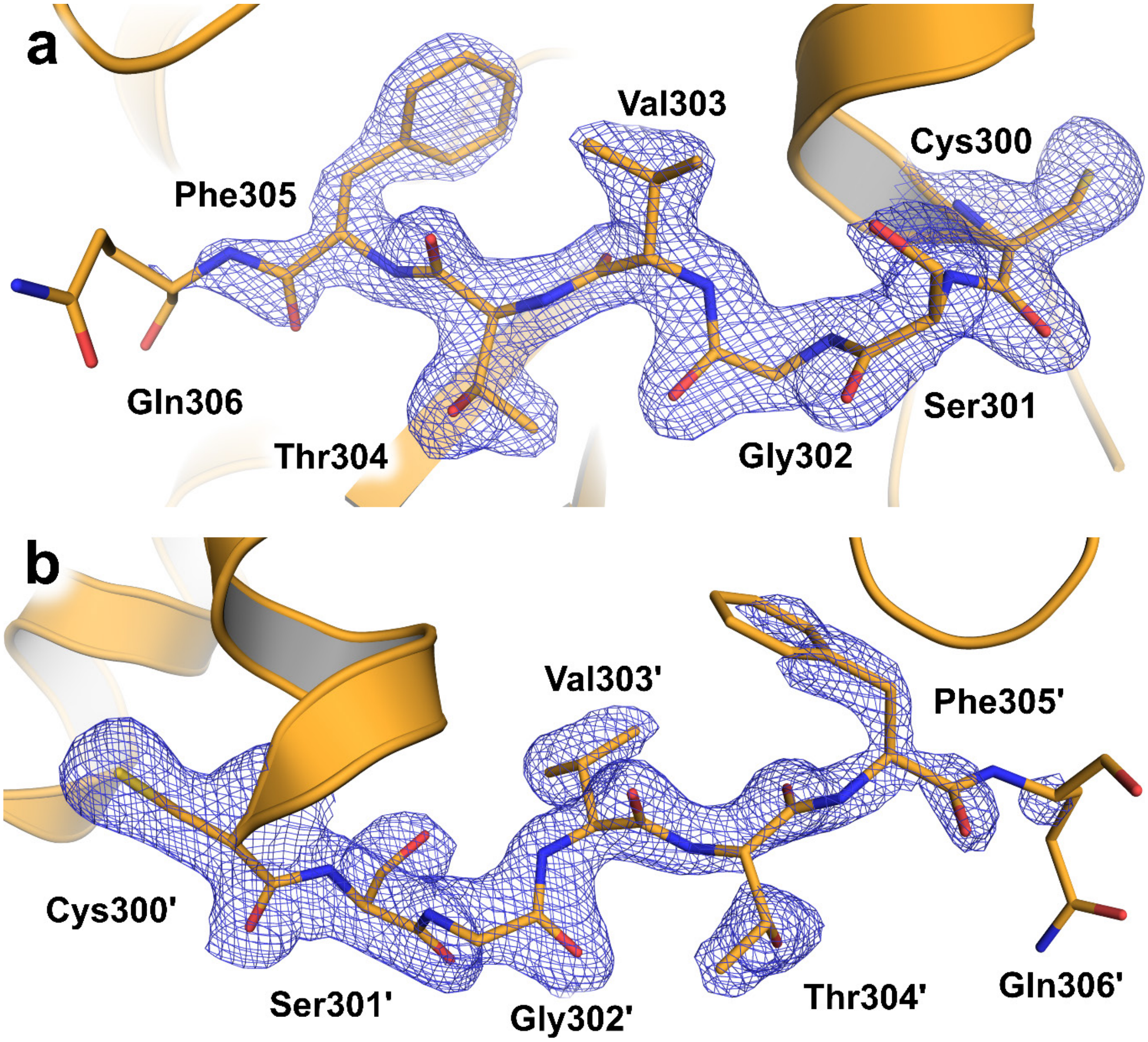

Figure S1: Electron density of the C-terminal residues in $M^{pro/C145A}$-Substrate structure.

C-terminal residues a) protomer 1 and b) protomer 2 as orange sticks with 2Fo-Fc electron density is shown as blue mesh contoured at 1σ.

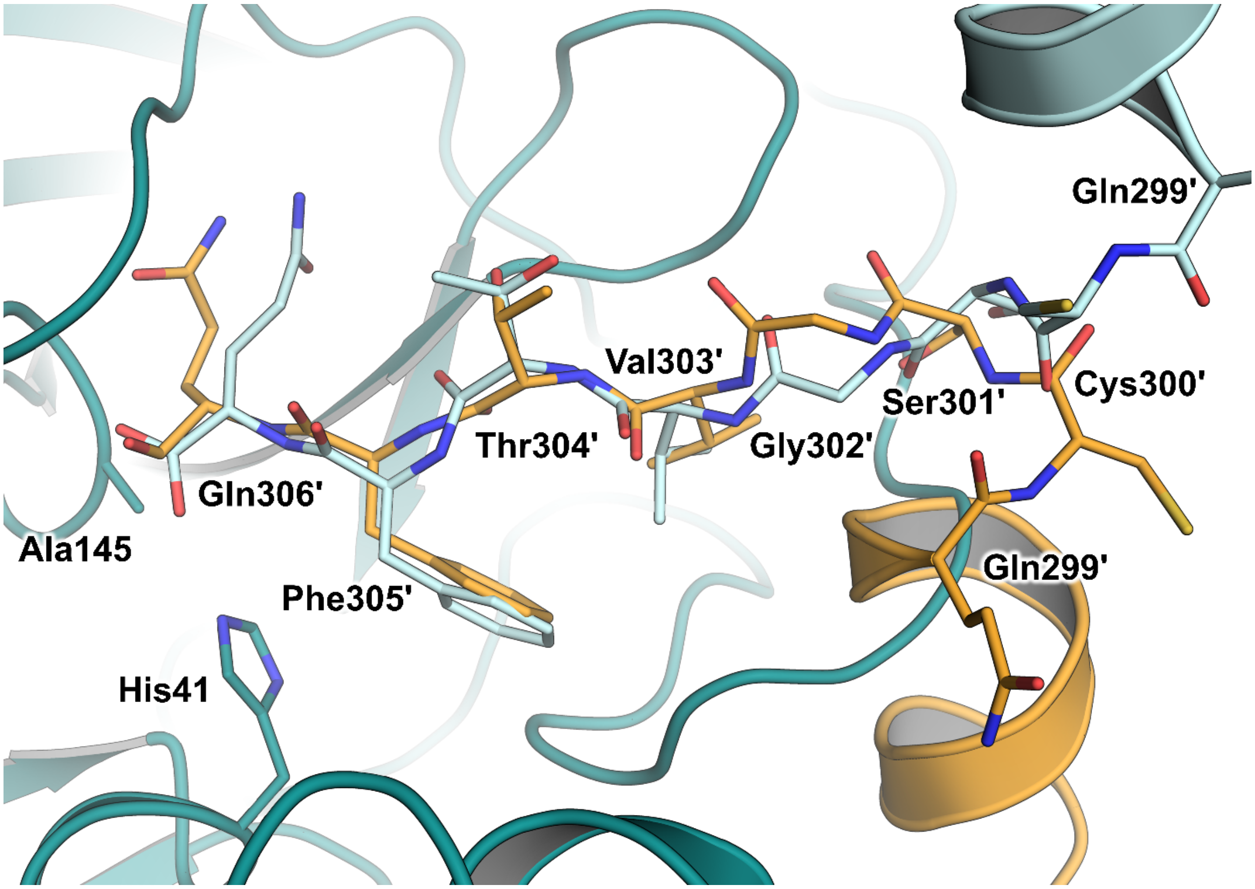

Figure S2. C-termini superposition of $M^{pro/C145A}$ -Substrate and $M^{pro/C145A}$ -Product complexes

C-terminal comparison showing the similarity between the C-termini of the $M^{pro/C145A}$ -Substrate co-crystal structure (orange carbons, 7N89) and the C-terminus autoprocessing complex mimic captured by Lee *et al.* 2020[1] (light cyan carbons with teal carbons for crystallographic symmetry mate, 7JOY). Superposition by least-squares-fit of main chain from residues 303-306 of 7N89 protomer 2 to 7JOY protomer 2.

References

(1) Lee, J., Worrall, L. J., Vuckovic, M., Rosell, F. I., Gentile, F., Ton, A.-T. T., Caveney, N. A., Ban, F., Cherkasov, A., Paetzel, M., and Strynadka, N. C. J. J. (2020) Crystallographic structure of wild-type SARS-CoV-2 main protease acyl-enzyme intermediate with physiological C-terminal autoprocessing site. *Nat. Commun. 11*, 5877.
